# Antibody Response to Influenza Hemagglutinin Conserved Stalk Domain after Sequential Immunization with Old Vaccine Strains

**DOI:** 10.1155/2024/5691673

**Published:** 2024-02-13

**Authors:** Alita Kongchanagul, Promsin Masrinoul, Chompunuch Boonarkart, Ornpreya Suptawiwat, Prasert Auewarakul

**Affiliations:** ^1^Center for Vaccine Development, Institute of Molecular Biosciences, Mahidol University, Salaya, Thailand; ^2^Department of Microbiology, Faculty of Medicine Siriraj Hospital, Mahidol University, Bangkok, Thailand; ^3^Center of Learning and Research in Celebration of HRH Princess Chulabhorn's 60th Birthday Anniversary, Chulabhorn Royal Academy, Bangkok, Thailand

## Abstract

Hemagglutinin (HA) is the major envelope glycoprotein and antigen on the surface of influenza virions. The glycoprotein comprises a globular head and a stalk region. While immunodominant epitopes on influenza HA head are highly variable, the stalk domain is conserved. The variability of the HA head causes the antigenic drift that made the requirement of annual update of vaccine strains. Induction of antibody against the stalk domain has been proposed as an approach for a broadly protective influenza vaccine strategy. Sequential exposure to influenza strains with highly diverse HA heads but conserved stalks have been shown to induce antibody to the low immunogenic stalk domain. Here, we tested this approach by using old influenza vaccine strains that are decades apart in evolution. Inactivated whole virion vaccine of influenza A/Puerto Rico/8/1934, A/USSR/92/1977, and A/Thailand/102/2009 (H1N1) was sequentially immunized into BALB/c mice in comparison to immunization using single strain (A/Thailand/102/2009 (H1N1)). The sequentially immunized mice developed higher levels of binding antibody to the stalk domain. These suggested that using old vaccine strains in sequential vaccination may be a possible approach to induce antibody to the conserved stalk domain.

## 1. Introduction

Seasonal influenza viruses evolve under a strong positive selection by the host immune response. This leads to frequent changes in the viral antigenic epitopes commonly referred to as the “antigenic drift” [1]. Because of this antigenic drift, the seasonal influenza vaccine is annually updated for its viral strain composition [2]. The antigenic drift is usually caused by changes in the immune dominant and variable epitopes on the globular head of the viral hemagglutinin (HA), which is the major envelope glycoprotein responsible for receptor binding [3, 4]. The HA is a trimeric protein comprising a variable globular head and a more conserved stalk domain [5]. The stalk is less immunogenic but can be a target of neutralizing antibody. The conserved epitopes on the HA stalk are an interesting target for the development of a broadly protective influenza vaccine [6]. Various strategies have been developed to induce protective antibody targeting this conserved stalk epitope, including chimeric HA and headless HA. The chimeric HA approach uses HA heads from avian influenza viruses and a stalk of seasonal influenza virus. Chimeric HA constructs with different heads are sequentially immunized so that only the stalk domain can induce anamnestic response, while the different heads can only induce a primary response in each immunization dose. The sequential vaccination with different immunogens that share only a common target epitope has been shown to be successful in animal studies. The concept was further supported by the observation that people infected or immunized with the 2009 H1N1 influenza virus developed antibody to the stalk domain. This was explained by an anamnestic response to the stalk, which was similar to the previously circulating seasonal H1N1 influenza, whereas the globular heads of the seasonal H1N1 and the 2009 pandemic H1N1 were too different for an anamnestic response [7–9]. Following the same line of thought, it was conceivable that sequential immunization with old vaccine strains, which are highly diverse in the globular head but have a similar stalk, may be able to induce antibody against the stalk domain. Using old vaccine strains and conventional vaccine technology has an advantage of having less safety concern and requiring no change in production technology. Therefore, we tested this approach in a mice model and show here that sequential immunization with old and distantly related seasonal H1N1 vaccine strains resulted in antibody response against the stalk domain. This approach may provide a viable immunization strategy to raise antibody responses against conserved epitopes of influenza HA for a broad protection.

## 2. Materials and Methods

### 2.1. Cells and Viruses

Madin–Darby canine kidney (MDCK) and human embryonic kidney (HEK) 293T cells were maintained in minimal essential medium and Dulbecco's modified Eagle's medium (DMEM) (both from Gibco). Each was supplemented with 10% fetal bovine serum (Gibco) and 100 units/ml of penicillin 100 *µ*g/ml of gentamycin. The MDCK cell line was kindly provided by M. Peiris, the University of Hong Kong, and the 293T cell line was obtained from ATCC and used at passage 20th and 12th, respectively.

Chimeric influenza A virus (cH9/1) was kindly obtained from Prof. Peter Palese. The chimeric HAs contain the globular head domain of A/guinea fowl/Hong Kong/WF10/99 (H9N2) HA and the common stalk domain from the A/Puerto Rico/8/1934 (H1N1) virus.

The recombinant influenza A virus used in this study was produced by the reverse genetics method as previously described [10]. Briefly, HEK293T and MDCK cell lines were mixed and cocultured in 2 ml of opti-MEM media (Gibco) in a 6-well plate for 18 hours. The cells were cotransfecting with pHw2000 plasmids carrying the cloned HA gene (A/Puerto Rico/8/1934, or A/Nonthaburi/102/2009 or A/USSR/92/1977) and the other 7 genes from the A/Puerto Rico/8/1934 (H1N1) (PR8) strain by using TranslT-LT1 transfection reagent (MirusBio, USA, Cat. No. MIR2300). At 30 hours posttransfection, 1 ml of MEM containing 2 *μ*g/ml of L-(tosylamido-2-phenyl) ethyl chloromethyl ketone (TPCK) treated trypsin (Sigma, USA) was added. The supernatant was sampled for virus on day 3 by hemagglutination assay. The culture was passaged in MDCK cells for two passages until high titer viral growth. After that, viruses were propagated in the 9-day-old embryonated eggs to obtain higher viral titer.

### 2.2. Inactivation and Purification of Viruses

Allantoic fluids (50 eggs per virus strain) were passed through a 0.22 *μ*m filter and inactivated with 0.4% formalin for 3 days at 4°C. Inactivated viruses were dialyzed to eliminate formalin, and virus inactivation was confirmed by inoculation into MDCK cells. Next, inactivated viruses were concentrated with 50% polyethylene glycol 8000 (PEG8000: Sigma), and the concentrated samples were purified using sucrose step gradient ultracentrifugation (bottom 55%; top 35%) in a Beckman MLS-50 rotor at 50,000 g for 2 hours. Fractions of 500 *μ*l were collected. HA proteins were analyzed by SDS-PAGE and western blotting, and fractions containing influenza viruses were pooled. Purified viruses were dialyzed to eliminate sucrose and concentrated with 50% PEG8000. The concentrated viruses were resuspended to homogeneity in PBS and stored at −80°C until tested.

### 2.3. Western Blotting

The purified viruses were loaded onto a 10% polyacrylamide gel. The gel was transferred onto a nitrocellulose membrane. The blot was probed with 1 : 100 of influenza anti-A/California/7/2009 (H1N1)v HA serum (NIBSC 09/152) as a primary antibody. HA proteins were detected with conjugate rabbit antisheep horseradish peroxidase (HRP; Invitrogen) and visualized with diaminobenzidine (DAB, Sigma). The HA protein band was compared for the intensity with a standard HA protein of the virus A/California/04/20009 (H1 HA protein with c-terminal histidine tag from influenza virus A/California/04/20009 recombinant from baculovirus; BEI resources NR-15749) with a gel documentation system (Syngene).

### 2.4. Animal

Female BALB/c mice between 6 and 8 weeks old were obtained from the National Laboratory Animal Center, Mahidol University. Experiments were performed in a biological safety cabinet in a BSL-2 facility, and the protocol was approved by the Faculty of Veterinary Science Animal Care and Use Committee (FVS-ACUC).

### 2.5. Animal Experimental Design

Mice were divided into 3 groups: homologous, heterologous, and control groups. Homologous-boosted mice (*n* = 10) were primed with the inactivated pandemic 2009 influenza vaccine and boosted at week 4 and 8 with the same vaccine strain. Heterologous-boosted mice (*n* = 10) were primed with the inactivated pandemic 2009 influenza vaccine and boosted with the inactivated A/Puerto Rico/8/1934 influenza vaccine at week 4 and the inactivated A/USSR/92/1977 influenza vaccine at week 8. The control group (*n* = 5) received PBS at the initial prime and at week 4 and 8. Mice were anesthetized by isoflurane inhalation. Ten *μ*g antigen in PBS mixed with AddaVax (Invitrogen) adjuvant at a 1 : 1 ratio in a volume of 50 *μ*l was administered intramuscularly for each immunization. Sera were collected 2 weeks after the third immunization. Mice were euthanized by overdose isoflurane inhalation. Blood was collected by direct cardiac puncture and allowed to clot for 1 hour at room temperature before centrifugation at 10,000 g for 15 minutes. Sera were stored at −20°C unit tested.

### 2.6. Serum Preparation

Serum samples were treated with a receptor-destroying enzyme (RDE: Denka Seiken, Tokyo, Japan) overnight at 37°C and heat-inactivated for 30 min at 56°C to eliminate the nonspecific inhibitors, serum complement, and inactivate RDE. The treated sera were diluted 1 : 10 before measuring the level of antibody production and neutralizing activity using enzyme-linked immunosorbent, microneutralization, and plaque-reduction neutralization assay.

### 2.7. Enzyme-Linked Immunosorbent Assay (ELISA)

Titers of anti-HA stalk domain antibodies in the sera were determined by enzyme-linked immunosorbent assay (ELISA). 100 ng of purified recombinant chimeric HAs (cH6/1 or cH9/1 HA) of the purified virus, diluted in 50 mM bicarbonate/carbonate coating buffer, was coated overnight onto each well of 96-well plate (Immulon 2; Nunc) at 4°C. Then, plates were washed twice with 0.025% tween/1X PBS (PBS-T) and blocked with 5% nonfat milk in 1X PBS for 30 minutes at RT. The serum was diluted serially in 5% nonfat milk, incubated for 1 hour at 37°C, and then washed 4 times with PBS-T. The serial dilution process started by placing 146 *μ*l of 1 : 100 diluted serum into column 1. Subsequently, 46 *μ*l of the diluted sample was serially transferred into 100 *μ*l of diluent in the next well. This resulted in a serial ½ log_10_ dilution. Goat antihuman IgG-horseradish peroxidase (HRP) (Meridian Life Science Inc.) as the secondary antibody was diluted 1 : 2000 in 5% nonfat milk; 50 *μ*l was added to each well, incubated for 1 hour at 37°C, and then washed 4 times with PBS-T. 50 *μ*l of TMB peroxidase substrate system (KPL, USA) was added into each well and incubated in the dark for 10 min at room temperature. 1M H_2_SO_4_ was added to stop the reaction, and the plate was read at OD 450/630 by ELISA plate reader.

### 2.8. Microneutralization (microNT) Assay

The treated sera were two-fold serially diluted in duplicate and incubated with the test virus at a final concentration of 100TCID50/100 *μ*l for 2 hours at 37°C. The serum-virus mixture was transferred onto an MDCK monolayer maintained in MEM supplemented with TPCK-treated trypsin for 24 hours. The cells were preseeded at 30,000 cells per well of a 96-well plate overnight before the assay. The cells were fixed with 80% acetone, and the level of viral infection was measured using a mouse-specific monoclonal antibody against influenza A nucleoprotein (Millipore, Temecula, USA) as the primary antibody and HRP-conjugated goat antimouse immunoglobulin (SouthernBiotech, Alabama, USA) as the secondary antibody. Finally, the signal of ELISA was developed with tetramethylbenzidine-H2O2 substrate (KPL, USA), and optical densities were measured at 450 nm. Antibody titer is defined as the reciprocal of the highest serum dilution that reduces ≥50% of the amount of viral nucleoprotein in the reaction wells as compared to the virus control wells.

### 2.9. Plaque-Reduction Neutralization Assay

The treated sera were two-fold serially diluted in duplicate and incubated with 30 to 50 PFU of virus for 2 hours at 37°C. The serum-virus mixture was inoculated on a monolayer of MDCK cells in a 12-well plate and incubated at 37°C for 2 hours with intermittent rocking every 15 minutes. After 2 hours of incubation, the cells were covered with an agar medium consisting of a mixture of 2% agarose (Promega, USA) and MEM supplemented with trypsin TPCK. At 2 days postinfection, the monolayer was fixed with 10% formalin for 1 hour and then stained with 1% crystal violet. Plaques were counted for each serum, and the percent inhibition was calculated versus the virus control.

### 2.10. Statistical Analysis

The comparison between groups was performed using an independent samples *t*-test and SPSS Statistics software (SPSS, Inc., Chicago, IL, USA). A *p* value ≤0.05 was taken as statistically significant.

## 3. Results

### 3.1. Selection of Old Vaccine Strains

**Figure 1:**
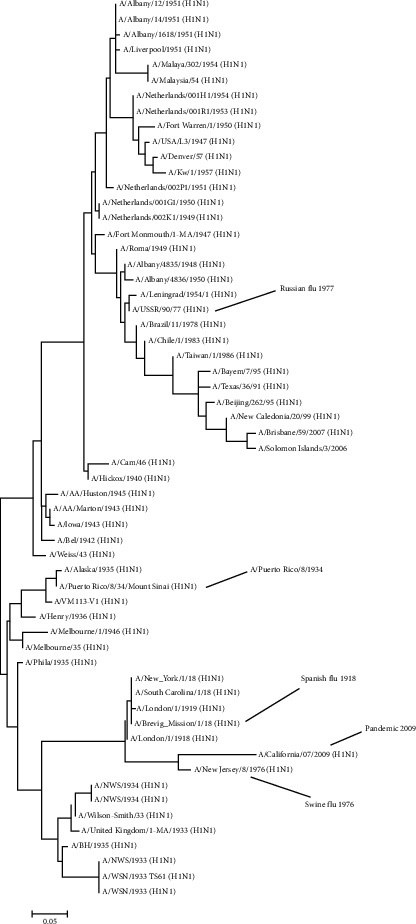
Maximum-likelihood tree of the H1 amino acid sequence shows the selected old vaccine strains on different branches.

**Figure 2:**
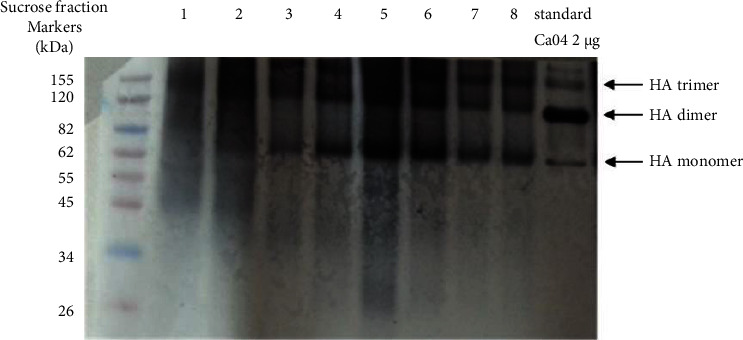
Western blotting of fractions from sucrose step gradient ultracentrifugation for purification of virion from the allantoic fluid. The blot was probed by an HA-specific sheep serum.

To obtain H1N1 influenza strains with only distantly related globular heads and highly conserved stalk domain, we performed phylogenetic analysis of pandemic and seasonal H1N1 influenza strains by maximum-likelihood method implemented in PAUP version 4.0 and selected 3 viruses that belong to different branches in the phylogenetic tree as shown in Figure 1. This selection aimed at having HAs with the most distances from one another in the history of H1 circulation in the human population. The three selected strains are A/Puerto Rico/8/1934, Russian flu 1977, and pandemic flu 2009. The amino acid similarities in the globular head and stalk domain among the tree strain are 83.6% and 97.0%, respectively. This indicates that the three strains are highly diverse in their globular head but very similar in their stalk domain. To obtain influenza HA antigen for the immunization, the reverse genetic viruses carrying the HA of A/Puerto Rico/8/1934, A/Nonthaburi/102/2009, or A/USSR/92/1977 viruses were grown in embryonated eggs. Allantoic fluid was harvested and checked for the titer of influenza virus by hemagglutination assay. The allantoic fluid yielded virus at hemagglutination titers of 32–4096/50 *μ*l. Whole virions were inactivated, concentrated, and purified from the harvested allantoic fluid and analyzed for the HA content by western blot after sucrose step gradient ultracentrifugation (Figure 2).

### 3.2. Antibody Response against the HA Globular Head

**Figure 3:**
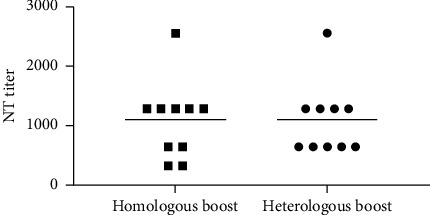
NT titers against A/Thailand/102/2009 virus in the homologous and heterologous immunized mice groups at week 10 after immunization.

To confirm that the immunization was effective, the antibody response against the homologous H1 strain was evaluated. The geometric mean hemagglutination inhibition and neutralization titers against A/Thailand/102/2009 were found to be 557 and 1114 in group 1 and 640 and 1114 in group 2 mice, respectively (Figure 3). This indicated that the immunized antigen was adequate, and the immunization was effective in inducing the normal antibody responses against the dominant globular head epitopes.

### 3.3. Antibody to the Stalk Domain in Mice Sequentially Immunized with Different HA Strains

**Figure 4:**
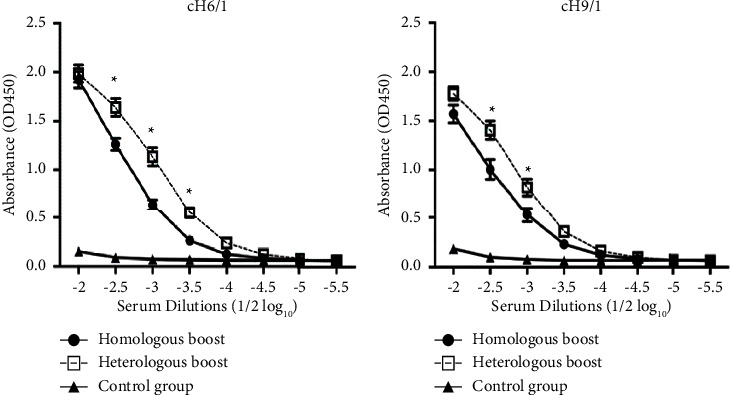
ELISA binding to the stalk domain of HA using purified recombinant chimeric HA; chimeric H6 head with H1 stalk (a) and chimeric H9 head with H1 stalk (b). Homologous-boosted mice (*n* = 10) were primed with inactivated pandemic 2009 influenza vaccine and boosted at week 4 and 8 with the same vaccine strain. Heterologous-boosted mice (*n* = 10) were primed with inactivated pandemic 2009 influenza vaccine and boosted with inactivated A/Puerto Rico/8/1934 influenza vaccine at week 4 and inactivated A/USSR/92/1977 influenza vaccine at week 8. The control group (*n* = 5) received PBS at the initial prime and at week 4 and 8 boost. The chimeric HAs were used to avoid binding to the HA globular head. Immunized mice did not have antibody response to H6 or H9 as the immunized antigens were H1. Therefore, the binding antibody that was detected in this ELISA belonged to the common HA stalk in the immunized and ELISA antigens. The asterisks indicate significant differences at *p* < 0.05 (*t* test).

After completing that vaccination, mice in both heterologous- and homologous-boosted groups showed binding antibody in an ELISA assay against cH6/1 and cH9/1 chimeric HA. The chimeric HAs were used to avoid binding to the HA globular head. Immunized mice should not have antibody response to H6 or H9 as the immunized antigens were H1. Therefore, the binding antibody that was detected in this ELISA belonged to the common HA stalk in the immunized and ELISA antigens. Since the globular head of the chimeric HAs was derived from unrelated H6 and H9 viruses, which have no cross-reactivity with the globular head of H1, the binding activity indicated reactivity of the mouse sera to the H1 stalk domain in the chimeric HA molecules. All the sera were shown to be negative for hemagglutination inhibition against H9. This confirmed the specificity of the ELISA signal to the stalk domain. These chimeric HAs have been previously used to detect the antibody to the stalk domain of the H1 in a number of studies [9]. Mice in the heterologous-boosted group showed a significantly higher binding signal in the ELISA assay (*t*-test, *p* < 0.05) as compared to the homologous-boosted group (Figure 4). This indicated that the heterologous boosts could induce higher antibody response against the common stalk domain.

**Figure 5:**
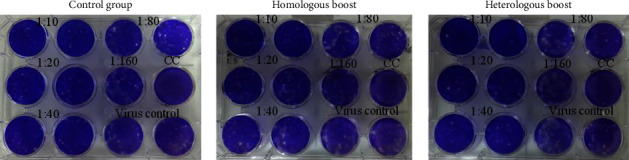
Plaque plates showing plaque-reduction neutralizing activity of sera from unimmunized control mice and immunized mice in homologous and heterologous groups. Plaque reduction was comparable in all groups, indicating the absence of neutralizing antibody against the HA stalk.

To find out whether the stalk-specific binding antibody was capable of neutralizing the virus, the sera were tested for plaque-reduction neutralization assay against reverse genetic viruses carrying the chimeric H9/1 HA. As a control for the stalk-specific neutralization, a monoclonal antibody known to target a neutralizing epitope on the stalk domain was used. The monoclonal antibody (MAb 6F12) was able to significantly reduce the plaque number of the cH9/1 virus at a concentration equal to or higher than 5 *μ*g/ml. In contrast, all the immunized mouse sera showed only a background level of neutralization activity similar to the nonimmunized mice (Figure 5). This suggested that the observed binding antibody against the stalk domain was either too low to neutralize the virus or targeted nonneutralizing epitopes in the stalk domain.

## 4. Discussion

Our data clearly showed a possibility of using old seasonal influenza vaccine strains in sequential immunization to induce an anamnestic response against the common HA stalk domain. The sequentially immunized mice developed binding antibody against the chimeric HAs carrying the H1 stalk. This ELISA technique has been previously used to demonstrate binding antibody to the stalk domain [9]. The globular heads of the chimeric HAs carried unrelated avian HA heads and were, therefore, not recognized by mice immunized with H1 HA, and the detected binding, therefore, belonged to stalk-binding antibody. Our data also demonstrated that the distances between the old vaccine HAs antigens representing 4–8 decades of evolution were sufficient to suppress the anamnestic response to the globular heads and to focus the response to the common stalk domain.

Previous studies showed that sequential heterologous immunization with HA antigens with similar stalk but different globular heads could induce stalk-specific antibody response [6]. An approach of using chimeric HAs with globular heads from avian influenza viruses combined with similar H1 stalk was tested in animal models and shown to be effective in inducing stalk-specific neutralizing antibody. The chimeric vaccine approach has entered clinical development with promising results [11]. These chimeric HAs are, however, new antigens that have never been used in humans, and a number of safety profiles are required to ensure safety in human use. In contrast, old H1N1 vaccine strains have the benefit of being antigens that have been used safely in humans. Reintroducing these old vaccine strains may have a lower regulatory requirement and make the development process less time- and resource-consuming. These H1N1 vaccine strains are highly conserved in their stalk region and thus should effectively provide the boost for stalk-specific antibody. The globular heads of these old vaccine strains are highly diverse across decades of antigenic drift and are unlikely to be effective in boosting antibody against highly variable epitopes. In contrast, current seasonal influenza vaccines use strains with small changes in the globular head, which induce effective anamnestic responses against variable epitopes and little responses against conserved stalk epitopes. Our study was conducted in naïve hosts, and it is conceivable that responses in humans previously exposed to seasonal influenza would be different. It is possible that older individuals may have immune memory to older viral strains and respond less effectively to the conserved stalk domain. On the other hand, young individuals who only have immune memory to the current H1N1 virus may readily respond to conserved epitopes upon immunization with an old strain. In comparison to the current approach with chimeric avian HA heads, our approach may also induce antibody responses against conserved epitopes on HA head. Our results suggested that the difference in the variable epitopes among the old vaccine strains was sufficient to allow a more effective response to conserved epitopes. Although the conserved stalk domain drew much interest in universal vaccine design, conserved epitopes in the HA1 head have been identified [12, 13]. It is, however, more difficult to isolate the conserved epitopes on HA head for vaccine design. Our approach may theoretically induce responses to such epitopes, and further studies are required to test this hypothesis.

Our results did not show a significant neutralizing antibody against the stalk domain. While neutralizing antibodies against the stalk epitopes have gained a lot of interest, it is still unclear whether they are required for protection, and other mechanisms such as antibody-dependent cellular cytotoxicity (ADCC) have been proposed to play an important role in the protection by stalk antibody [14]. It is also possible that better immunogen preparation and formulation will result in a better neutralizing response against the conserved stalk epitopes.

## Data Availability

The data used to support the findings of this study are included within the article.
